# Preliminary Effectiveness of a One-Week Summer Day Camp for Improving Children’s Health Behaviors and Psychosocial Well-Being Outcomes

**DOI:** 10.3390/children11091097

**Published:** 2024-09-07

**Authors:** Qiaoyin Tan, Yuxin Nie, Paul Son, Renee A. Underwood, Peyton Murray, Callie Hebert, K-Lynn McKey, Chelsea Hendrick, Amanda E. Staiano, Senlin Chen

**Affiliations:** 1 School of Kinesiology, Louisiana State University, Baton Rouge, LA 70803, USA; qtan1@lsu.edu (Q.T.); ynie4@lsu.edu (Y.N.); dson2@lsu.edu (P.S.); 2 Pediatric Obesity and Health Behavior Laboratory, Pennington Biomedical Research Center, Baton Rouge, LA 70808, USA; renee.underwood@selu.edu (R.A.U.); peyton.murray@pbrc.edu (P.M.); callie.hebert@pbrc.edu (C.H.); chelsea.hendrick@pbrc.edu (C.H.); amanda.staiano@pbrc.edu (A.E.S.); 3 Department of Kinesiology and Health Studies, Southeastern Louisiana University, Hammond, LA 70402, USA; 4 School of Kinesiology, University of Louisiana Lafayette, Lafayette, LA 70506, USA; k-lynn.mckey@louisiana.edu

**Keywords:** childhood obesity, health disparity, healthy living, intervention

## Abstract

Purpose: Summer day camp offers children opportunities to grow knowledge and skills, be physically active, and have fun. Compared to healthy children, at-risk children (i.e., overweight, or with obesity and chronic health conditions) typically display less optimal health behaviors and psychosocial well-being, especially during summer months. This study examined the preliminary effectiveness of an American-Diabetes-Association-sponsored summer day camp at improving children’s health behaviors (i.e., physical activity, screen time, diet, sleep) and psychosocial well-being outcomes (i.e., quality of life [QoL], enjoyment, weight-related self-efficacy). Method: The sample consisted of 39 participants, including 19 boys and 20 girls, with majority being overweight (*n* = 4 or 10%) or with obesity (*n* = 26 or 67%), who attended the day camp for one week. Results: Significant improvements were observed in screen time, quality of life, and physical function. The results further showed significant time by gender interaction effect for overall QoL (*p* < 0.05, $\eta_{p}^{2}$ = 0.15), physical health (a dimension of QoL; *p* < 0.05, $\eta_{p}^{2}$ = 0.18), and significant time by household income for the psychosocial health (another dimension of QoL), favoring boys and those from higher income families. Discussion: The findings indicate a positive preliminary effectiveness of the summer camp at reducing children’s screen time and improving their QoL, especially in boys and those from higher-income families. Future research should focus on health disparities and expansion of this camp for the potential of longer-term and more robust effects related to wellness, nutrition literacy, physical activity promotion, and obesity prevention.

## 1. Introduction

Southern states in the United States (e.g., Louisiana) have higher obesity prevalence than the national average [1]. Obesity is a common comorbidity of other chronic health conditions, such as diabetes, hypertension, cardiovascular diseases, metabolic syndromes, and mental health disorders [2]. The root cause of obesity is complex and varies from person to person, as numerous heredity and environmental factors collectively influence weight fluctuation [3]. However, weight status can be effectively managed via lifestyle modification by achieving a caloric balance between intake (e.g., eating) and expenditure (e.g., physical activity, sedentary behavior) [4,5,6]. Obesity interventions should target younger populations, so youth may build the competence, confidence, and behaviors needed for healthy living both physically and mentally from childhood onward [7]. 

Prior research has shown that children frequently gain excess weight during summer months when they do not attend school or receive structured education [8,9,10,11]. Obesity risk is typically higher among children from low socioeconomic status (SES) households and those who are already overweight or with other health conditions [12,13]. During summer break, camps offer children opportunities to receive continued education, adult supervision, and relevant hands-on skill-building experience, which may subsequently help them grow knowledge and skills, be physically active, and have fun [12,14]. However, many summer camps are privately run, demanding costly admission tuitions, parent time, and private transportation, which are more affordable for affluent families. In contrast, children from low SES households are constrained by limited resources, hence reduced accessibility to summer camps. During summer break (>10 weeks), these children are likely to be at home with minimal parent supervision, excessive screen time, irregular sleep schedule, and unhealthy eating behaviors [15], jeopardizing their overall health and well-being. 

Summer months may be perceived, by the child, as long and boring when there is no access to meaningful programs, resources, and opportunities outside of the home. This especially applies to at-risk children who are overweight and/or with chronic health conditions (e.g., diabetes). Compared to healthy children, those at-risk may display less optimal health behaviors and psychosocial well-being. Psychosocial well-being refers to the state of mental, emotional, and social health of an individual [16]. For optimal well-being, children are recommended to (1) participate in 60 min of moderate-to-vigorous physical activity (MVPA); (2) watch two hours or less of screen time; (3) have balanced meals in moderation; and (4) sleep 9–11 h per day [17]. Meeting these recommendations regularly contributes to children’s healthy living habits, which are associated with positive health outcomes. In this study, we focused on children’s health behaviors (i.e., physical activity, screen time, diet, sleep) and psychosocial well-being, including health-related quality of life (QoL; overall and specific to physical, cognitive, affective, and social functions [18]), physical activity enjoyment, and weight-related self-efficacy. These factors are important variables for children to develop and maintain positive health (e.g., a healthy weight status). Additionally, physical activity enjoyment, weight-related self-efficacy, and health behaviors are relevant factors when evaluating the feasibility and impact of a summer camp for at-risk children. However, few prior studies have extensively examined children’s summer camp experience and its potential in influencing health behaviors and psychosocial well-being (QoL, enjoyment, self-efficacy) [19,20].

The primary aim of this study was to examine the preliminary effectiveness of an *American Diabetes Association* (ADA)-sponsored summer camp (i.e., *Project Power*), in improving at-risk children’s health behaviors (i.e., physical activity, screen time, diet, sleep) and psychosocial well-being outcomes (i.e., QoL, enjoyment, weight-related self-efficacy) [21]. The ADA-sponsored summer camp was previously tested over a 5-day period at a different location (in San Antonio, Texas) and shown to improve health knowledge and increase self-reported and parent-reported physical activity engagement [22]. The ADA selected Baton Rouge, Louisiana, as a high-risk location for pediatric type 2 diabetes, to expand Project Power and test its effectiveness on multiple domains of children’s health. Our secondary aim was to explore the pre-to-post-test changes in health behaviors and psychosocial well-being by gender (boys vs. girls), household income (<$50,000 annually vs. ≥$50,000 annually), and weight status (normal weight, overweight, obesity).

## 2. Methods

### 2.1. Research Design, Setting, and Participants

Following recommendations in feasibility research [23], this pilot study employed a single group pre-to-post-test design to examine the preliminary effectiveness of *Project Power*. Two cohorts of participants, aged 6 to 14 years old (M = 9.69 ± 1.56), attended the summer camp over two summers (2017 and 2018) held at the Pennington Biomedical Research Center (PBRC) in Baton Rouge, Louisiana. Participants were recruited based on their risk for type 2 diabetes using their current weight status and family medical history (at the time of recruitment). Twelve participants were enrolled in the first summer, and 27 were enrolled in the second summer. Both iterations (2017 and 2018) received the same program. The PBRC Institutional Review Board approved the study protocol (#2017-014). All participants were offered the opportunity to participate in the research study, and study participation was not a requirement to attend the camp. Parental consent and participant assent were obtained in writing from all study participants. The camp was free to participants, and participants did not receive any additional compensation for taking part in the study.

The sample consisted of 19 boys and 20 girls, with majority being White/Caucasian (*n* = 16) or Black/African American (*n* = 15). Of the sample, 18 were from low SES households (<$50,000 annual household income) and 17 from higher SES households (≥$50,000 annual household income). The majority of the participants attended all five camp sessions (*n* = 33, 84.6%). Most participants were either overweight (BMI percentile = 85–94.9%: *n* = 4 or 10%) or obese (BMI percentile = 95% or higher: *n* = 25 or 67%). Nine participants were at a normal weight (BMI percentile = 5–84.9%; *n* = 9 or 23%). 

### 2.2. Project Power

*Project Power*, an initiative by the American Diabetes Association (ADA), originally began as a program known as “Power Up” and was later rebranded as Project Power. The program is designed to help both children and adults reduce their risk of developing type 2 diabetes through education, increased physical activity, and healthy lifestyle choices. It was a one-week summer day camp for children aged 6 to 14 who had obesity or were at risk for type 2 diabetes. The camp adapted a comprehensive program called the *Kids N Fitness*© lifestyle program, which provided participants with interactive nutrition education, structured physical activity sessions, and behavior modification strategies. The camp was held on the campus of Pennington Biomedical Research Center, provided healthy lunches and snacks prepared by dietitians, and ran from 9 a.m. to 4 p.m. Monday to Friday for 1 week in June or July. The daily schedule included morning physical activities, midday nutrition education, afternoon games, and behavioral strategy sessions. Sessions were primarily held in a large auditorium and classrooms, and physical activity was performed on fields and at a nearby community park and recreational facility within walking distance. The campers also visited the local farmer’s market that sells fresh produce and meats on Thursday mornings on campus. The camp counselors included undergraduate students majoring in Kinesiology, overseen by a physical education teacher, and the nutrition sessions were led by a registered dietitian. A registered nurse was available for children who had special medical needs such as medications to take during the day or medical contraindications to exercise. Community volunteers assisted in various activities, and a nurse was available for medical situations. Nutrition education involved cooking demonstrations, interactive games, quizzes, and discussions to teach balanced meals, portion control, and healthy food choices. The physical activities consisted of both inside and outside exercise, sports, games, and skill themes, to encourage active living, enhance health, and teamwork. These are common activities that students experience in physical education classes in the United States. They may be played after school or at home, although most of them are best organized as group activities. In addition, the behavior modification strategies involved teaching the participants goal-setting, self-monitoring, positive reinforcement, and problem-solving skills to develop sustainable healthy habits. Parents were highly encouraged to attend the parent sessions held in two afternoons directly after the day camp and were also provided with handouts detailing activities and information to reinforce healthy behaviors at home. 

### 2.3. Data Collection

Data collection occurred onsite at PBRC. During the orientation session or in the morning of Day 1 of the summer camp (baseline) and then in the afternoon of Day 5 (post-test), trained data collectors (1) administered hard copy questionnaires to the participants to measure their health behaviors and psychosocial well-being and administered a baseline demographics survey to the parents and (2) measured the participants’ body weight and height to determine weight status. 

### 2.4. Variables and Measures

Health Behaviors: The participants’ health behaviors, including physical activity, screen time (i.e., time spent on TV, videos and computer games, respectively), and sleep (i.e., hours and minutes of sleep) were measured using a validated survey [24]. To measure physical activity, participants were asked the number of days in past 7 days they were physically active for 60 min or more (choices ranged from 0 to 7). This question was adopted from the *CDC’s Youth Risk Behavior Surveillance System* (YRBSS) [24]. This physical activity question has been previously used to measure children and adolescents’ physical activity with acceptable validity and reliability [25]. Also related to physical activity, participants reported the number of days they participated in physical activity outside of their home. In addition, participants were asked to report their screen-time use (in hours and minutes) for both TV viewing and playing video or computer games, as well as their sleep duration, using questions adapted from the U.S. National Health and Examination Survey. 

Quality of Life (QoL): *The Pediatric Quality of Life Scale* (PedsQL) was used to evaluate health-related QoL of participants. PedsQL has acceptable reliability and validity in diverse populations, with internal consistencies ranging from 0.70 to 0.90 [26]. The PedsQL includes a core scale and other specific modules, which capture children’s physical, emotional, social, and school functions. The questionnaire consists of 23 items, divided into four dimensions: physical function (8 items), emotional function (5 items), social function (5 items), and school function (5 items). Following the original scoring guideline, the *Psychosocial Health* summary score was aggregated by summing up the items for the emotional, social, and school function scales; while the *Physical Health* summary score was expressed by the sum of physical function items. According to the scoring guidelines, higher PedsQL scores represent higher health-related QoL and vice versa [27].

Physical Activity Enjoyment: The participants’ enjoyment of physical activity was measured using the *Physical Activity Enjoyment Scale* (PACES) [28]. The scale has a Cronbach’s alpha of 0.89 and is a validated instrument designed to assess how much children enjoy participating in physical activities. PACES has been used in prior research and has shown consistent psychometric characteristics [29]. PACES consists of 16 items, which are scored according to a 5-point Likert scale, ranging from “disagree a lot” to “agree a lot”. The questionnaire includes statements such as “I enjoy it” and “It’s no fun at all”, where participants rate their level of agreement or disagreement. For example: “I enjoy it”: 1 (disagree a lot) to 5 (agree a lot). The scores were summed to provide an overall enjoyment score, with higher scores indicating greater enjoyment of physical activities.

Weight-related self-efficacy: The participants’ weight-related self-efficacy was assessed using the *Weight Efficacy Lifestyle Questionnaire* (WEL), which measures an individual’s confidence in managing their weight across different situations. The WEL scale has shown high internal consistency, with Cronbach’s alpha being 0.90 in various populations. The validity and reliability of the WEL has been established through extensive research, and the scale has been used in various populations [30]. The WEL consists of eight items, with a scale of 10, ranging from 0 (not confident) to 9 (very confident). The questionnaire assesses confidence in situations such as “resisting eating when feeling anxious” and “controlling overeating when there is a lot of food available”. e.g., “I can resist eating when I am anxious or nervous”, “I can control overeating when there is a lot of food available”, 0 (Not confident) to 9 (Very confident). The scores were summed to provide a total self-efficacy score, with a higher total score representing higher confidence in managing weight in the face of challenges [30].

Anthropometric measures: Each participant was measured in private for height and weight wearing light clothing. Height was measured to the nearest 1.0 cm using a portable stadiometer. Weight was measured to the nearest 0.1 kg using a high-precision electronic scale. Body mass index (BMI) z-score and BMI percentile (BMIp) score were calculated based on the participant’s age, sex, height, and weight, in reference to the *2000 CDC Growth Charts* [31].

Sociodemographic factors: Parents completed a paper survey at study orientation or when dropping off their child on the first day of camp to report sociodemographic characteristics including sex, date of birth, parents’ marital status, highest level of education, family income, occupation, and employment status, as well as brief medical history. In addition, the participants’ attendance of the parent sessions (offered two afternoons during the 1-week day camp) was recorded.

### 2.5. Data Analysis

We used SPSS 29 to conduct paired *t*-tests to analyze any pre-to-post-test changes in health behaviors and psychosocial well-being outcomes. Cohen’s *d* was computed as effect size to interpret the between time differences. Subsequently, we conducted repeated measure multivariate analyses of variance (RM-MANOVAs) to examine the pre-to-post-test changes in health behaviors and psychosocial well-being outcomes by gender (boys vs. girls) and household income ($50,000 as the split value). We further explored the pre-to-post-test changes in the outcomes (using gain scores: post-test–pretest data) by weight status (normal weight, overweight, obesity) but did not perform inferential statistical analysis due to the uneven sample sizes between the weight groups. Descriptive results, including both raw and estimated marginal means, were obtained to quantify and visualize group differences. Partial eta square ($\eta_{p}^{2}$) was reported as effect size from results from the RM-MANOVAs, while significance level was set to be α = 0.05.

## 3. Results

Table 1 reports descriptive results for body height, weight, health behaviors (sleep, screen time, physical activity), and psychosocial well-being outcomes at the two measurement time points (baseline and post-test) for the camp sessions (2017 and 2018) combined. Statistically significant improvements were observed in screen time (*t* = −2.10, *p* < 0.05, *d* = −0.43), total QoL (*t* = 2.52, *p* < 0.05, *d* = 0.37), and physical health of QoL (*t* = 2.93, *p* < 0.01, *d* = 0.67), with moderate effect sizes favoring the post-test over baseline results. Changes in the other outcome variables were marginal and not statistically significant (*p* > 0.05, Cohen’s *d* < 0.30). These results indicate some positive overall impact of the *Project Power* to the participants. We report, in the next section, the results by weight status, gender, and household income, to identify intervention impact on specific groups.

Table 2 below shows the pre-to-post-test changes (post-test—pre-test scores) in health behaviors and psychosocial well-being by weight status group (normal weight, overweight, obesity) for the camp sessions (2017 and 2018) combined. Note, the final sample reduced from 39 to 33, due to participants withdrawal or missing post-test data. Descriptive results revealed some between-group differential patterns. For example, compared to participants with normal weight, participants with obesity showed more favorable pre-to-post-test changes in screen time (i.e., total screen time, video/computer games), time spent outside, PA enjoyment, QoL, and weight-related self-efficacy. Children with normal weight showed slightly more favorable changes in TV viewing and PA behavior (i.e., # of active days). However, because of the uneven sample size across the three groups, inferential statistical analyses were not performed.

**Figure 1:**
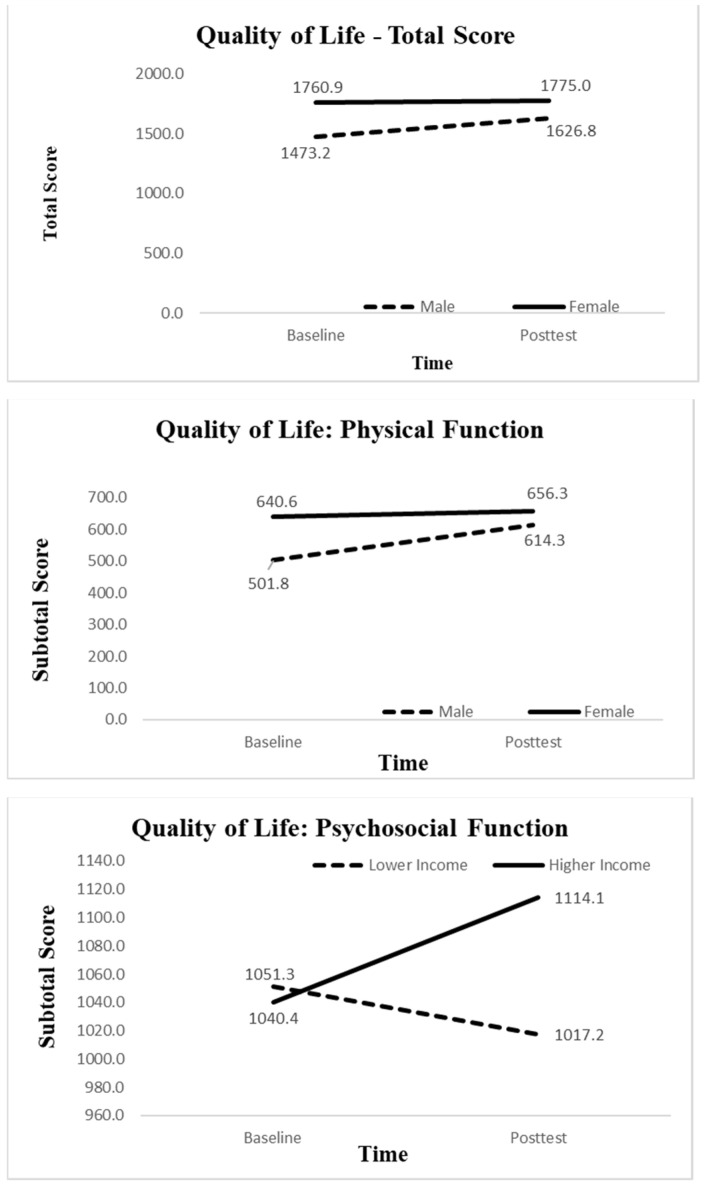
Pre-to-post-test changes in health-related QoL by gender or household income level. Top panel: total QoL. Middle panel: physical health dimension of QoL. Bottom panel: psychosocial health dimension of QoL.

The RM-MANOVAs verified that screen time (*F*_1,26_ = 4.80, *p* < 0.05, $\eta_{p}^{2}$ = 0.16), overall QoL (*F*_1,26_ = 6.46, *p* < 0.05, $\eta_{p}^{2}$ = 0.20), and the physical health of QoL (*F*_1,26_ = 9.64, *p* < 0.01, $\eta_{p}^{2}$ = 0.27) were the only three outcome variables that showed statistically significant pre-to-post-test changes. More importantly, the RM-MANOVAs further demonstrated a significant *time by gender* interaction effect for total QoL (*F*_1,26_ = 4.47, *p* < 0.05, $\eta_{p}^{2}$ = 0.15) and physical health (*F*_1,26_ = 5.51, *p* < 0.05, $\eta_{p}^{2}$ = 0.18) and a significant *time by household income* for psychosocial health, favoring boys and those from higher income families. Figure 1 below illustrates these significant changes in QoL scores (total QoL, physical health, or psychosocial health) across the groups. 

## 4. Discussion

The purpose of this study was to test the preliminary effectiveness of *Project Power* on participant’s health behavior and psychosocial well-being. We observed significant pre-to-post-test changes in screen time and QoL among the overall sample and favorable results among specific groups, including participants with obesity, boys, and those from higher income families. These findings are discussed below.

The camp demonstrated preliminary effectiveness in improving the campers’ screen time and QoL. Children and adolescents spend on average over five hours per day in front of electronic screens (computer, tablets, smartphone, etc.) with most of this screen time accumulated while sitting [32]. Excessive sedentary screen time is detrimental to children’s health and contributes to psychological disorders and issues (distress, anxiety, aggressive and antisocial behaviors) [33]. Reducing children’s screen time, especially during summer break, is crucial and should be regarded as a priority by parents [32]. The reduction in screen time (*d* = 0.43), including TV viewing and playing video and computer games, found in this current study is encouraging. Similarly, the significant change in total QoL and the physical health dimension of QoL further supports the preliminary effectiveness of the camp among the priority population. These findings show that health-focused summer programs can effectively promote healthy lifestyles and improve the health level of high-risk adolescents.

When examining pre-to-post-test changes by weight status, we observed participants with obesity showed significant improvement in most of the outcome variables, including screen time (i.e., total screen time, video/computer games), time spent outside, physical activity enjoyment, QoL, and weight-related self-efficacy. These results suggest that *Project Power* had greater preliminary effectiveness in benefitting participants with obesity (vs. those with normal weight). Upon the immediate end of the camp, participants with obesity improved their weight-related self-efficacy and physical activity enjoyment, two important determinants of physical-activity behavior. Children with obesity struggle with self-esteem and often lack the self-efficacy to overcome obstacles and barriers, to be physically active, and to control their dietary behaviors [34]. Improving weight-related self-efficacy and having an enjoyable physical activity experience may enable success in the face of obstacles and barriers. Behaviorally, participants with obesity reported increased physical activity (not as much as those with normal weight) and time spent outdoors (greater increase than normal weight group) and reduced their total screen time. Furthermore, favorable changes in QoL (total and its two dimensions) further reinforce the utility of *Project Power* in benefitting psychosocial well-being. 

Lastly, our results unraveled significant pre-to-post-test changes in QoL by gender and household income, favoring boys and those from higher SES households. Specifically, boys reported much lower QoL than girls at baseline, but upon attending the camp, their QoL significantly improved, while girls’ QoL remained largely unchanged. By household income, in the pre-test, participants from both groups reported similar psychosocial health. However, in the post-test, the higher SES group reported increased QoL, while the lower SES group reported decreased QoL. These group differences highlight the need for a tailored approach to ensure boys and girls from various socioeconomic backgrounds can benefit equally from summer camps such as *Project Power*.

One strength of this study was the focus on a vulnerable group ─ children at risk for type 2 diabetes (77% overweight or with obesity) at a critically important but often overlooked period (i.e., summer break). Prior research has rarely examined the feasibility and preliminary effectiveness of summer camps in improving children’s health behaviors and psychosocial well-being [35]. Another study strength was the use of validated instruments to assess health behaviors and psychosocial well-being outcomes. We acknowledge several limitations. Although the pre-experimental design was appropriate for assessing the preliminary effectiveness of the intervention as a feasibility study, future research aiming for full effectiveness should incorporate a randomized controlled trial design. Consequently, the findings related to effectiveness should be interpreted cautiously, focusing on feasibility rather than definitive outcomes. Additionally, the study faced challenges due to the small sample size and the brief duration of the intervention. These limitations arose from budget constraints and the availability of resources to operate Project Power, compounded by the timing of the camp’s sign-up, which occurred shortly before the summer break. Although the camp was centrally located in Baton Rouge, parents had to arrange transportation for their children, and the 9 a.m. to 4 p.m. schedule may not have been convenient for families with conflicting work obligations. Similarly, our study examined the acute effect of the summer camp; therefore, we did not collect data to inform the sustained effect of the intervention. Future research should include follow-up assessments to determine if the benefits observed immediately after the camp persist.

## 5. Conclusions

This study offers initial evidence that the Project Power summer camp can effectively enhance health behaviors and psychosocial well-being among children, particularly those susceptible to obesity and type 2 diabetes. Notable decreases in screen time and improvements in health-related QoL highlight the potential of short-term, focused interventions in promoting healthier lifestyles in young people. The findings of this short-term feasibility study contribute to the burgeoning literature on utilizing summer break as an opportunity to provide physically active, health-promoting time for children to build knowledge and skills, particularly for children at risk of chronic disease (obesity, diabetes). The significant improvements in screen time and health-related QoL substantiate the feasibility of the *Project Power* in reducing obesity and diabetes risks. The differences observed by gender and income groups emphasize the necessity of advocating inclusive and adaptive strategies to ensure that all children would benefit equally from the camp intervention. Future interventions should test an extended time frame of intervention delivery to make more sustained impacts on children’s behavior, with a consideration of the content, deliveries, and experiences to address the needs of campers from both boys and girls from all socioeconomic backgrounds, regardless of their weight or health status. Future research should also consider recruiting a larger and more diverse sample, with a randomly assigned control group, to verify the impact of the *Project Power*, when delivered via a summer camp [7,14].

## Figures and Tables

**Table 1:** Descriptive results for the study variables at baseline and post-test.

Variables	Baseline (Orientation or Day 1)			Post-Test (Day 5)			*d*
	*N*	*M*	*SD*	*N*	*M*	*SD*	
Body Height (cm)	39	143.94	9.16	-	-	-	-
Body Weight (kg)	34	51.88	19.09	34	51.71	19.01	−0.01
Sleep Duration (hour)	33	9.87	1.61	33	9.49	1.82	−0.21
Screen Time: Total (hour)	33	5.45	2.68	33	4.48	2.22	−0.43 *
Screen Time: TV (hour)	33	2.94	1.80	33	2.36	1.73	−0.32
Screen Time: Video and Computer Games (hour)	33	2.52	1.91	33	2.12	1.63	−0.22
Time Outside (hour)	33	2.79	1.80	33	3.15	1.87	0.19
# of Active Days (days)	33	4.06	2.32	33	4.18	2.19	0.06
Physical Activity Enjoyment	33	61.82	9.37	33	62.06	9.18	0.03
Total Quality of Life	33	1638.64	365.08	33	1721.97	318.67	0.37 *
Physical Health	33	580.30	158.45	33	638.64	122.17	0.67 **
Psychosocial Health	33	1058.33	225.84	33	1083.33	225.40	0.16
Weight Self-Efficacy	33	52.73	19.53	33	54.82	19.90	0.14

Note. * denotes significant change (*p* < 0.05). ** denotes very significant change (*p* < 0.01).

**Table 2:** Pre-to-post-test changes (gain scores) in the variables by weight status.

Variables	Normal Weight (*n* = 8)		Overweight (*n* = 4)		Obesity (*n* = 21)	
	*M*	*SD*	*M*	*SD*	*M*	*SD*
Sleep Duration (hour)	0.63	1.04	−1.88	3.66	−0.48	1.63
Screen Time: Total (hour)	−0.88	2.10	0.00	1.83	−1.19	2.99
Screen Time: TV (hour)	−1.00	1.31	0.00	1.41	−0.52	2.06
Screen Time: Video and Computer Games (hour)	0.13	1.55	0.00	0.82	−0.67	1.65
Time Outside (hour)	−0.25	1.49	0.75	1.50	0.52	1.72
# of Active Days (days)	0.88	2.36	−1.50	3.11	0.14	2.83
Physical Activity Enjoyment	0.13	6.27	−7.00	5.48	1.67	8.73
Total Quality of Life	3.13	150.26	12.50	136.17	127.38	203.55
Physical Health	−515.63	206.99	−512.50	158.77	−365.48	164.22
Psychosocial Health	−25.00	114.17	31.25	59.07	42.86	149.82
Weight Self-Efficacy	−8.13	14.20	3.00	8.33	5.81	10.92

## Data Availability

The datasets presented in this article are not readily available unless a data-sharing agreement is established between the research team and the request individuals or party, upon the Pennington Biomedical Research Center IRB’s review and approval. Requests to access the datasets should be directed to the Project Power PI Amanda Staiano (amanda.staiano@pbrc.edu).
